# Association between metabolites in tryptophan-kynurenine pathway and inflammatory bowel disease: a two-sample Mendelian randomization

**DOI:** 10.1038/s41598-023-50990-9

**Published:** 2024-01-02

**Authors:** Fangqian Yu, Yutong Du, Cong Li, Haiyan Zhang, Weiming Lai, Sheng Li, Zhenhao Ye, Wenbin Fu, Shumin Li, Xiang-Guang Li, Ding Luo

**Affiliations:** 1 The Second Affiliated Hospital of Guangzhou University of Chinese Medicine, Guangzhou, 510120 China; 2 Department of Pharmaceutical Engineering, School of Biomedical and Pharmaceutical Sciences, Guangdong University of Technology, Guangzhou, 510006 China; 3 Liuzhou Workers’ Hospital, The Fourth Affiliated Hospital of Guangxi Medical University, Liuzhou, 545000 China

**Keywords:** Immunological disorders, Immunology, Microbiology, Diseases, Gastroenterology

## Abstract

Previous observational studies have suggested an association between tryptophan (TRP)–kynurenine (KYN) pathway and inflammatory bowel disease (IBD). However, whether there is a causal relationship among them remains unclear. Therefore, a two-sample Mendelian randomization (MR) study was conducted to explore the potential causal effects of crucial metabolites in TRP–KYN pathway on IBD and its subtypes. Using summary data from genome-wide association studies, a two-sample MR was employed to evaluate the genetic associations between TRP and KYN as exposures and IBD as an outcome. The inverse variance weighted method was used as the primary MR analysis, with MR-Egger, weighted mode, simple mode, and weighted median methods as complementary analyses. The odds ratios (OR) and 95% confidence intervals (CI) were determined for TRP–IBD (OR 0.739, 95% CI [0.697; 0.783]), TRP–UC (OR 0.875, 95% CI [0.814; 0.942]), TRP–CD (OR 0.685, 95% CI [0.613; 0.765]), KYN–IBD (OR 4.406, 95% CI [2.247; 8.641]), KYN–UC (OR 2.578, 95% CI [1.368; 4.858], and KYN–CD (OR 13.516, 95% CI [4.919; 37.134]). Collectively, the MR analysis demonstrated a significant protective association between TRP and IBD, whereas KYN was identified as a risk factor for IBD.

## Introduction

Inflammatory bowel disease (IBD) is a group of immune-mediated disorders that predominantly affect the gastrointestinal tract, including ulcerative colitis (UC) and Crohn’s disease (CD). These disorders are commonly associated with clinical manifestations such as abdominal pain, diarrhea, mucopurulent bloody stool, and other symptoms. In severe cases, IBD can result in malnutrition and intestinal perforation^1^. This chronic condition is currently incurable and necessitates lifelong medication. The global prevalence rate of IBD exceeds 0.3%^2^. The impact of IBD on patients’ quality of life is significant, and its high prevalence represents a substantial economic and medical burden to society. Therefore, investigating the risk factors and pathogenesis is of paramount importance for IBD.

Tryptophan (TRP) is an important component of the human diet and plays a crucial role in inflammatory responses and gastrointestinal health^3^. TRP deficiency can lead to dysbiosis of the gut microbiota, resulting in gastrointestinal and even systemic inflammation^3,4^. In the human body, the major catabolic pathway of TRP is the kynurenine (KYN) pathway (KP), which accounts for 95% of total TRP degradation^5^. TRP can regulate intestinal inflammation^6–8^ and thus affect the occurrence of inflammatory bowel disease (IBD). Clinical studies have found that the serum TRP levels in IBD patients are significantly lower than those in normal control individuals, and there is a significant increase in the KYN/TRP ratio^9^. Furthermore, these levels are closely related to the degree of endoscopic inflammation and disease activity^10,11^. TRP deficiency and metabolic abnormalities may promote the development of IBD^9^. Previous studies have also indicated the crucial role of TRP–KYN metabolism dysregulation in the occurrence and progression of IBD^12^. In gene expression research, current studies have revealed the involvement of several gene loci associated with the TRP–KYN metabolic pathway in the susceptibility and progression of inflammatory bowel disease (IBD). Through genome-wide association studies, polymorphisms in key gene loci, including IL23R^13^, NOD2/CARD15^14^, and ATG16L1^13^, are significantly associated with the risk of developing IBD. These gene loci are functionally linked to the TRP–KYN metabolic pathway, and their encoded proteins play critical roles in immune response regulation, inflammation signaling pathways, and autophagy, among other processes^15–18^. The association between TRP–KYN metabolites and IBD, however, could be biased due to confounding variables^19,20^, and the genetic and causal relationships are still unclear. Additionally, most of the above studies are observational and may have potential detection errors and confounding factors, which may reverse causality.

Therefore, we employed the Mendelian randomization (MR), a method of causal inference using genetic variation, to exclude genetic and causal relationships between crucial metabolites in TRP–KYN pathway and IBD. By utilizing the invariance of individual genotypes and Mendelian laws of inheritance (random allocation of alleles during gamete formation), it can avoid interference from common confounding factors, such as the postnatal environment, socioeconomic status, and behavioral habits^21^.

## Materials and methods

### Study design

This study investigated the causal relationship between TRP–KYN metabolites as exposures and the outcome of IBD by adhering to the fundamental principles and core assumptions of MR. Three assumptions required for MR analysis were met: (1) the genetic instrumental variables (IVs) used in the study were strongly associated with TRP and KYN; (2) the genetic IVs were not associated with any confounding factors related to IBD; and (3) the genetic IVs influenced IBD only through their effect on TRP and KYN. This study utilized publicly available datasets that had already received ethical approval and informed consent; therefore, additional ethical approval or informed consent was unnecessary.

### Data sources

We utilized the most up-to-date and comprehensive Genome-Wide Association Studies (GWAS) datasets currently available for investigating metabolites in TRP–KYN pathway and IBD. The GWAS summary statistics for IBD (including CD and UC) were obtained from The IEU GWAS database (https://gwas.mrcieu.ac.uk/datasets/), while the data for metabolites in TRP–KYN pathway (only TRP and KYN were available) were obtained from the GWAS catalog (https://www.ebi.ac.uk/gwas/). All of these datasets can be downloaded. To prevent bias, only individuals of European origin were included in this Mendelian randomization study. Tables S1 summarizes the study population, including the number of genetic variants (i.e., SNPs available, Ncase) used in the analysis (Table 1).

**Table 1:** Characteristics of the study population.

Phenotype	GWAS ID	SNPs available	Ncase	Ncontrol	Year published	Population
IBD	finn-b-K11_IBD_STRICT	16,380,455	3753	210,300	2021	European
UC	finn-b-ULCERNAS	16,380,457	2207	210,300	2021	European
CD	finn-b-K11_KELACROHN	16,380,466	940	217,852	2021	European
TRP^22^	–	15,430,214	8299	–	2022	European
KYN^23^	–	2,545,666	7824	–	2014	European

### Selection of genetic IVs

We implemented strict quality control criteria to filter the SNPs from the GWAS summary data. SNPs significantly associated with the risk factor at the genome-wide level (*p* value < 5 × 10^8^) were identified based on prior GWAS standards^24^. Subsequently, SNPs demonstrating independent inheritance and minimal linkage disequilibrium (LD) (r^2^ < 0.001, kb = 10,000) were selected. Ambiguous or palindromic SNPs were further excluded. To avoid the risk of weak instrumental bias, the F statistic (F = beta^2^/se^2^) was performed to evaluate the strength of the IV^25,26^. When F > 10, the association between the IV and exposures was deemed to be sufficiently robust, thereby safeguarding the results of the MR analysis against potential weak instrumental bias.

To measure the strength of the IVs, we calculated the F-statistic for each SNP, with those SNPs having an F-statistic < 10 being excluded as weak instruments. The MR-PRESSO test was further performed to detect and exclude any SNPs with potential pleiotropy^27^ and PhenoScanner (http://www.phenoscanner.medschl.cam.ac.uk/) was introduced to identify and remove SNPs with potential associations with confounding factors that might violate the independence assumption.

After several rounds of rigorous filtering, a set of eligible instrumental variables for the subsequent MR analysis were obtained.

### Statistical analysis

To examine the relationship between exposures and outcome, multiple MR approaches were employed. The inverse variance weighted (IVW) method was the primary approach used, given its ability to produce unbiased estimates and avoid confounding factors in the absence of horizontal pleiotropy^28^. Moreover, the MR-Egger, weighted mode, simple mode, and weighted median methods were also used for supplementary and substitution analysis^29^. To ensure the quality and robustness of our research results, we conducted various analyses, including pleiotropy, heterogeneity, and sensitivity analyses. MR-Egger regression was used to assess the presence of horizontal pleiotropy. Cochran’s Q-test statistic was used to examine heterogeneity among all SNPs in each database^30^. Finally, a leave-one-out sensitivity analysis was conducted to verify the stability of the results. The analyses were conducted using RStudio software version 4.2.2 and the packages ‘TwoSampleMR’ and ‘MRPRESSO’.

## Results

To investigate the causative influence of TRP on IBD risk, significant at the genome-wide level (*p* < 5 × 10^−8^) and independently inherited (r^2^ < 0.001, kb = 10,000) from the 15,430,214 SNPs, 173 SNPs were tentatively selected as IVs for IBD. Based on the findings from the PhenoScanner database, we excluded 28 SNPs associated with schizophrenia due to their potential impact on IBD occurrence^31^, such as rs12211045. Finally, a total of 145 SNPs were approved for MR analysis to assess the causal impact of TRP on IBD risk. Using the same methodology, we identified a total of 43 SNPs strongly associated with kynurenine (KYN) from a pool of 2,545,666 SNPs. Additionally, we excluded 6 SNPs associated with risk factors for IBD, including smoking (rs10774625, rs11065987, rs17630235, rs11066188), celiac disease (rs3184504, rs653178), and IBD (rs3184504, rs653178)^32–34^. Taking into account the differential effects of smoking on UC and CD^33,35^, we conducted MR analysis ultimately using 41 SNPs to assess the causal influence of KYN on UC risk, and 37 SNPs to investigate the causal effects of KYN on IBD and CD risk. All of the IVs had F-statistics > 10 (ranging from 29.76 to 39.70 for TRP and ranging from 29.58 to 111.13 for KYN). Supplementary Tables S1 contain comprehensive information regarding all the IVs.

### Causal effects of TRP on IBD, UC, and CD

**Figure 1:**
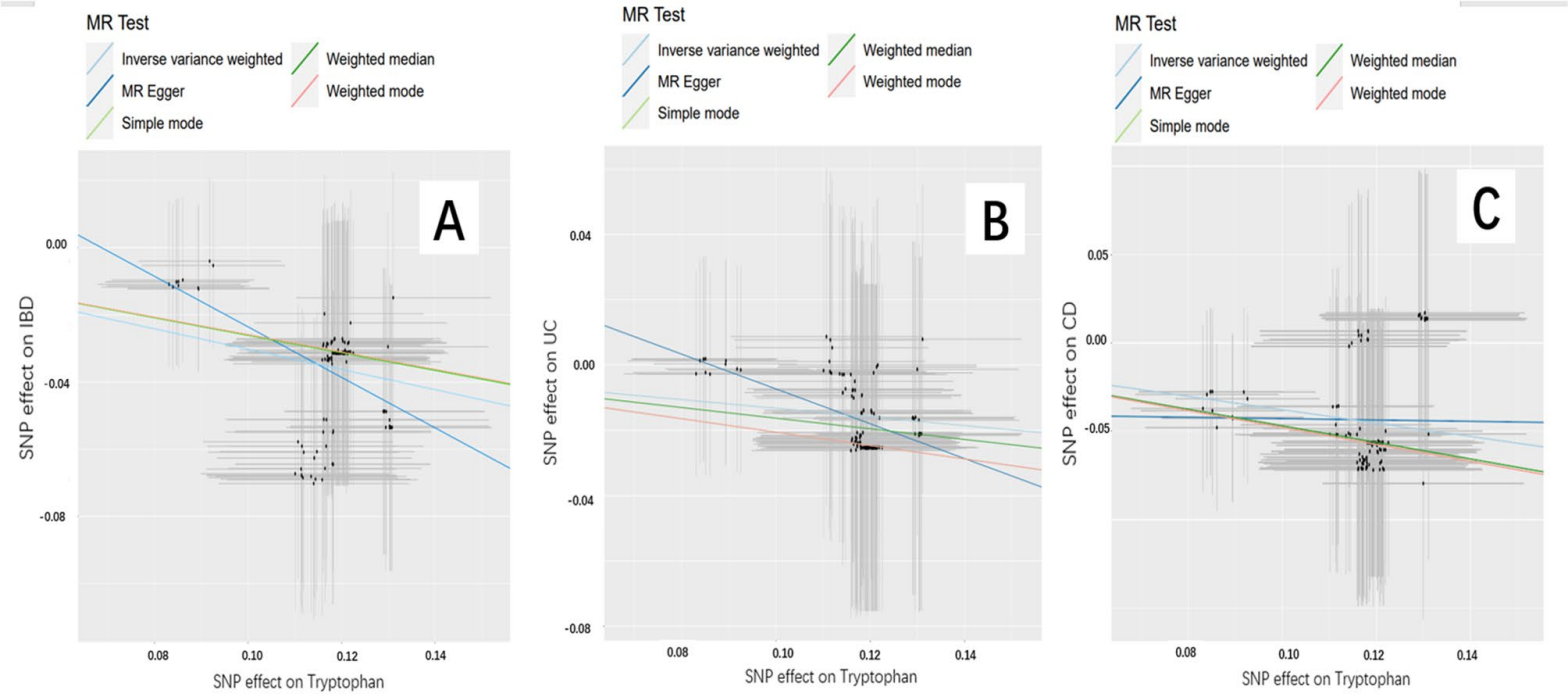
Scatter plots of the genetic associations with TRP against IBD risk using different MR methods. (**A**) TRP (tryptophan) against IBD (inflammatory bowel disease) risk, (**B**) TRP (tryptophan) against UC (ulcerative colitis) risk, and (**C**) TRP (tryptophan) against CD (Crohn’s disease) risk. The slopes of each line represent the causal association for each method.

The Mendelian randomization analysis demonstrated a significant association between TRP and IBD outcomes (Fig. 1), indicating that TRP has a protective effect against IBD (odds ratio [OR]_IVW_ = 0.739, 95% confidence interval (CI) [0.697–0.783]; *P* < 0.05), including CD ([OR]_IVW_ = 0.685, 95% CI [0.613–0.765]; *P* < 0.05) and UC ([OR]_IVW_ = 0.875, 95% CI [0.814–0.942]; *P* < 0.05). These results were consistent across IVW, MR-Egger, weighted median, simple mode, and weighted mode methods. Table 2 shows the details of the MR analysis investigating the causal effects of genetically predicted TRP on IBD, UC, and CD.

**Table 2:** Mendelian randomization estimates for causal effects of TRP on IBD.

Exposure	Outcome	Method	OR	95% CI	*P* value
TRP	IBD	IVW	0.739	0.697–0.783	1.19e−24
		MR-Egger	0.471	0.295–0.753	2.09e−03
		Weighted median	0.770	0.713–0.831	2.10e−11
		Simple mode	0.771	0.625–0.952	1.72e−02
		Weighted mode	0.771	0.635–0.938	1.04e−02
	UC	IVW	0.875	0.814–0.942	3.95e−04
		MR-Egger	0.586	0.323–1.063	8.13e−02
		Weighted median	0.849	0.774–0.932	6.17e−04
		Simple mode	0.814	0.644–1.028	8.68e−02
		Weighted mode	0.814	0.636–1.041	1.04e−01
	CD	IVW	0.685	0.613–0.765	2.56e−11
		MR-Egger	0.962	0.390–2.373	9.34e−01
		Weighted median	0.625	0.539–0.725	5.87e−10
		Simple mode	0.619	0.421–0.912	1.67e−02
		Weighted mode	0.619	0.420–0.913	1.71e−02

*IBD* inflammatory bowel disease, *UC* ulcerative colitis, *CD* Crohn’s disease, *TRY* tryptophan.

### Causal effects of KYN on IBD, UC, and CD

**Figure 2:**
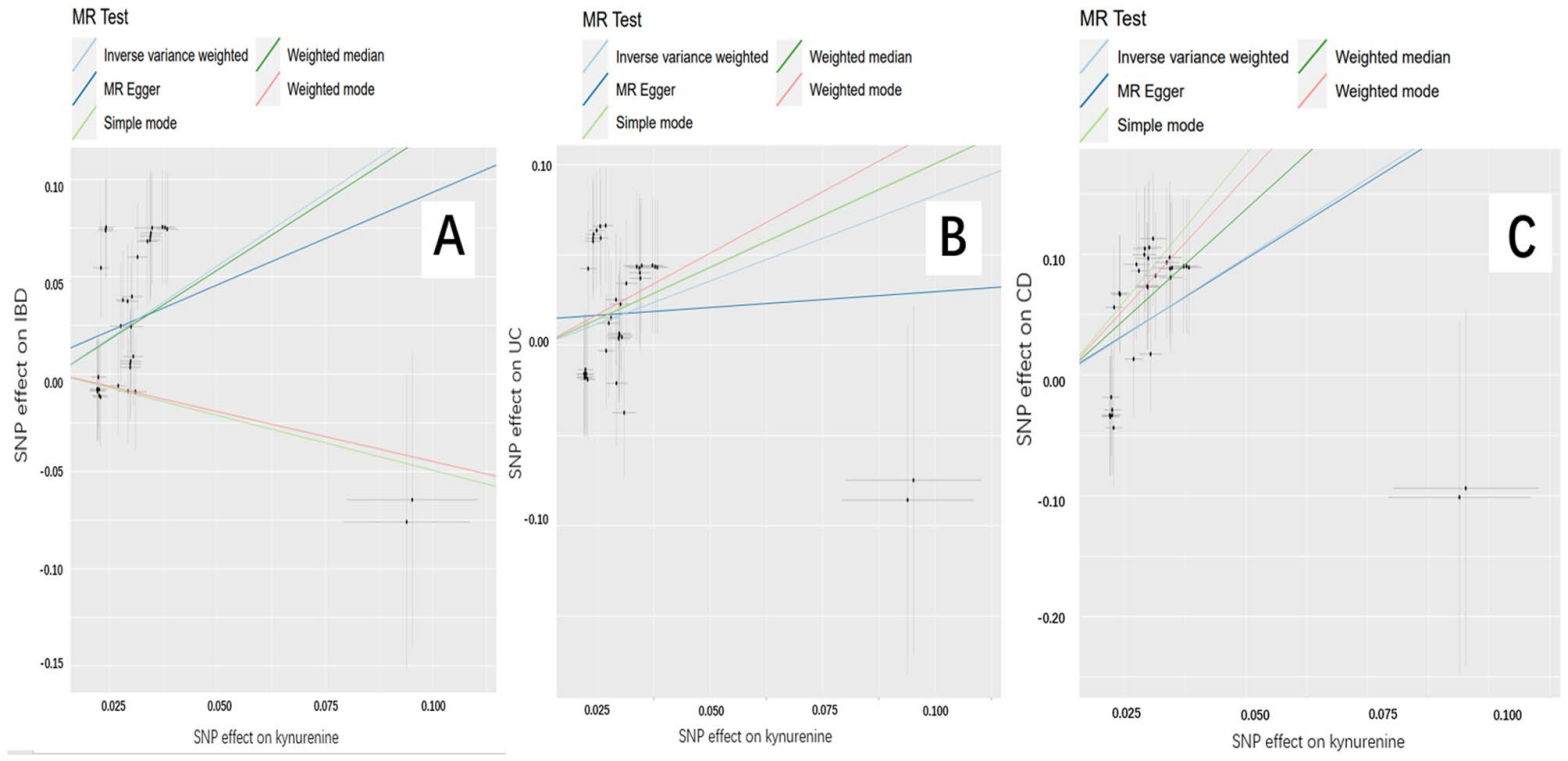
Scatter plots of the genetic associations with KYN(Kynurenine) against IBD (inflammatory bowel disease) risk using different MR methods. (**A**) KYN (Kynurenine) against IBD risk, (**B**) KYN (Kynurenine) against UC (ulcerative colitis) risk, and (**C**) KYN (Kynurenine) against CD (Crohn’s disease) risk. The slopes of each line represent the causal association for each method.

Figure 2 shows the results of estimating the causal effect of KYN on IBD, UC and CD. The MR results from the IVW showed a significant correlation between KYN and an increased risk of IBD ([OR]_IVW_ = 4.406, 95% CI [2.247–8.641]; *P* < 0.05).There was a relationship between KYN and UC, with OR > 1 and *P* < 0.05 in the IVW, indicating that KYN is a risk factor for UC ([OR]_IVW_ = 2.578, 95% CI [1.368–4.858]; *P* < 0.05).All MR methods showed a significant increase in the risk of CD with KYN ([OR]_IVW_ = 13.516, 95% CI [4.919–37.134]; *P* < 0.05). Details of the MR analysis investigating the causal effects of genetically predicted KYN on IBD (including UC and CD) are provided in Table 3.

**Table 3:** Mendelian randomization estimates for causal effects of KYN on IBD.

Exposure	Outcome	Method	OR	95% CI	*P* value
KYN	IBD	IVW	4.406	2.247–8.641	1.58 e−05
		MR-Egger	2.586	0.558–11.986	2.32 e−01
		Weighted median	4.163	1.767–9.808	1.10 e−03
		Simple mode	0.568	0.048–6.682	6.56 e−01
		Weighted mode	0.599	0.037–9.559	7.19 e−01
	UC	IVW	2.578	1.368–4.858	3.38 e−03
		MR-Egger	1.190	0.270–5.237	8.18 e−01
		Weighted median	3.156	1.249–7.976	1.50 e−02
		Simple mode	3.156	0.427–23.315	2.66 e−01
		Weighted mode	3.895	0.687–22.074	0.13 e−01
	CD	IVW	13.516	4.919–37.134	4.42 e−07
		MR-Egger	12.443	1.221–126.765	4.03 e−02
		Weighted median	38.857	9.166–164.720	6.81 e−07
		Simple mode	131.837	7.579–2293.046	1.90 e−03
		Weighted mode	79.732	4.190–1517.157	6.11 e−03

*IBD* inflammatory bowel disease, *UC* ulcerative colitis, *CD* Crohn’s disease, *KYN* kynurenine.

### Sensitivity analyses

To ensure the validity of the aforementioned results, we conducted additional analyses to assess pleiotropy, heterogeneity, and sensitivity. Assessment using the MR-Egger intercept and the MR–PRESSO global test showed no evidence of horizontal pleiotropy (all *P* > 0.05). As statistical heterogeneity was detected for KYN in IBD (*p* < 0.05), we employed the IVW approach in a random-effects model. Heterogeneity tests using MR-Egger and IVW methods for the remaining results did not reveal significant heterogeneity. Finally, our leave-one-out sensitivity analysis confirmed the robustness of our causal estimates of the effect of genetically predicted TRP on IBD. The results of the sensitivity analysis are presented in Table 4 and Supplementary Figs. S1–S6.

**Table 4:** Heterogeneity and pleiotropy analysis of TRP and KYN with IBD, UC and CD.

Exposure	Outcome	MR-Egger				IVW	
		Intercept	Pleiotropy	Cochran’s Q	Heterogeneity	Cochran’s Q	Heterogeneity
			*p* value	Statistic	*p* value	Statistic	*p* value
TRP	IBD	0.051	0.060	16.817	1	20.406	1
	UC	0.046	0.185	3.665	1	5.437	1
	CD	− 0.039	0.457	16.951	1	17.506	1
KYN	IBD	0.012	0.452	59.876	5.50e−3	60.861	5.93e−3
	UC	0.014	0.264	36.081	0.603	37.361	0.589
	CD	0.001	0.938	36.929	0.379	36.935	0.452

*IBD* inflammatory bowel disease, *UC* ulcerative colitis, *CD* Crohn’s disease, *TRY* tryptophan, *KYN* kynurenine.

## Discussion

This study provides a comprehensive analysis of the causal relationships between TRP–KYN pathway and IBD using summary GWAS data. Due to a lack of GWAS data for all metabolites in this pathway in public databases, we opted to focus our research on GWAS data specifically for the key metabolites in this pathway, namely TRP and KYN.

By performing MR analysis, we can circumvent cumbersome and confounder-prone steps to estimate causal relationships between exposure and outcome, providing genetic support for the physiological and pathological processes between TRP and IBD. Our MR analysis revealed that TRP is a protective factor for both UC and CD, while KYN is a risk factor for IBD. These findings were consistent across different Mendelian tools and statistical models, and no evidence of horizontal pleiotropy was detected in any of the analyses. Although there was some heterogeneity observed in one result, we utilized statistical methods to optimize our analysis, ensuring that our conclusions are reliable.

TRP is an essential amino acid in mammals and is a biosynthetic precursor of numerous microbial and host metabolites. It is mainly metabolized through the KYN, serotonin, and indole pathways^36^, with 95% of TRP being metabolized via the KYN pathway under the action of rate-limiting enzymes, such as indoleamine 2,3-dioxygenase (IDO) and tryptophan 2,3-dioxygenase (TDO)^5^. IDO is a key enzyme in the metabolism of TRP, and IDO directs TRP toward KYN degradation. Additionally, increased IDO activity reduces the availability of TRP for other pathways^37^. IDO activity can also be stimulated by inflammatory cytokines, leading to increased consumption of TRP, especially in inflammatory states^38^.

In patients with IBD, there are significant alterations in the levels of TRP and KYN. Numerous clinical studies have shown that the expression of IDO, the concentration of KYN, and the KYN/TRP ratio are higher in the colonic and ileal lesions of IBD patients than in those of normal control individuals^39^. These indices are positively correlated with disease severity^40^. However, in nonlesion tissue of IBD patients, these indices are lower and similar to those of healthy control individuals^41^. In addition, the concentration of KYN in serum samples of IBD patients is also significantly increased^42,43^. Steroids, salicylates, and antitumor necrosis factor (TNF) biologics, such as infliximab, significantly reduce IDO expression and reverse the changes in KYN concentration and the KYN/TRP ratio in the treatment of IBD^12,19,40,41^.

Previous studies have found that TRP and its metabolites regulate intestinal inflammation^9^ and may exert anti-inflammatory effects through mechanisms such as regulating cellular immune function^6^, regulating the homeostasis of the intestinal microbiota^8^, and maintaining the balance and stability of the intestinal mucosa^7,44^. For example, KYN and indole can activate the aryl hydrocarbon receptor (AhR) by binding to it in a series of processes known as the TRP-AhR pathway. The activated TRP-AhR pathway can induce the expression of downstream cytokines such as interleukin-22 (IL-22) and interleukin-17 (IL-17)^45^, regulate T-cell proliferation, and thereby regulate intestinal immunity^46^. The TRP-AhR pathway plays an important role in regulating intestinal inflammation^12^, and Card9, as a susceptibility gene for inflammatory bowel disease (IBD), is closely related to this pathway. Studies have found that the intestinal microbiota of Card9 gene knockout mice cannot metabolize TRP into AhR ligands, resulting in reduced production of IL-22 and increased susceptibility to colitis^47^. When Card9 gene knockout mice were inoculated with three strains of lactobacilli that can metabolize TRP or treated with an AhR agonist, intestinal inflammation in mice was reduced^8^. Researchers have fed wild-type and AhR gene knockout mice diets containing either no TRP or 0.5% TRP. They found that the TRP diet improved colitis symptoms and severity in wild-type mice but not in AhR gene knockout mice. The TRP diet reduced the expression of inflammatory cytokines in the wild-type group and increased the expression of IL-22 and Stat3 mRNA in the colon, which protected the integrity of the epithelium^48^. In a mouse colitis model induced by dextran sulfate sodium (DSS), mice that lacked a TRP diet showed significantly increased susceptibility to inflammation and decreased levels of antimicrobial peptides in the gut^49^, which could be improved by adding TRP. In a mouse model of DSS-induced colitis, mice in the TRP group had less weight loss, reduced frequency of bloody stools, and improved histological changes in colonic tissue compared to mice in the no-TRP diet group^50^. In a piglet model of DSS-induced colitis, adding extra TRP to their regular diet improved clinical symptoms of colitis, increased piglet weight, and reduced intestinal permeability, which was possibly related to the reduced expression of proinflammatory cytokines (tumor necrosis factor-α, interleukin (IL)-6, interferon (IFN)-γ) and apoptosis initiators (caspase-8, Bax)^51^. A reduction in AhR ligand production has also been observed in the microbiota of IBD individuals, and a TRP-rich diet can improve treatment by increasing AhR ligands^52^, which ensures normal gut metabolism^46,53^. These results suggest that supplementation with TRP can improve intestinal inflammation and regulate epithelial homeostasis. Thus, TRP may be an effective immunomodulator for the treatment of IBD.

Current research indicates that KYN has a protective effect on intestinal inflammation through the AhR pathway^53,54^, which contradicts our research findings that suggest that KYN may be a potential risk factor for IBD. Some researchers have suggested that the activation of the KYN pathway during an inflammatory state is part of a physiological negative feedback compensation mechanism aimed at counteracting disease symptoms^19,55^. In summary, the potential mechanisms underlying the relationships among TRP, KYN, and IBD are complex and require further investigation. Based on current research, changes in TRP and KYN levels may make the KYN/TRP ratio a potential surrogate marker for disease activity in IBD patients, which could aid in predicting treatment response or relapse and better monitoring of the disease^54^.

Targeted therapy and modulation of the TRP–KYN pathway have primarily been the focus of cancer research^56^. Regulating key enzymes of TRP metabolism, such as IDO and TDO, can have therapeutic effects on diseases. In colorectal cancer cells, overexpression of IDO inhibits the infiltration of immune cells (CD3+ T, CD8+ T, CD3+ CD8+ T, and CD57+ NK cells), leading to immune escape, distant metastasis, consumption of local TRP, and the production of pro-apoptotic factors, significantly promoting disease progression and shortening overall survival of patients^57–59^. The IDO inhibitor 1-L-methyltryptophan (1-L-MT) reduces the transcription and proliferation of human colorectal cancer cells, induces mitochondrial damage, and causes cancer cell apoptosis^60^. When combined with chemotherapy and targeted drugs, 1-L-MT improves the overall survival rate of cancer patients^61^. Our research has revealed a clear causal relationship between TRP and KYN and IBD. We believe that further investigation of the mechanism between TRP–KYN metabolism and IBD may have great potential for targeted therapy of IBD.

Given the above, our study has several strengths. First, we used a large-scale GWAS dataset for MR analysis, allowing us to conduct robust MR analyses with a population of individuals of European ancestry, thus minimizing the impact of population stratification bias. Second, we used independent and strong genetic variants as instrumental variables (IVs) to mitigate the effects of linkage disequilibrium (LD) and weak instrument bias. Third, we used multiple MR methods, providing robust support for exploring the causal effects of TRP and KYN on IBD.

However, our study also has several limitations. First, although we used a GWAS dataset, we were unable to analyze different stages of IBD (e.g., active disease vs. remission) due to a lack of relative studies. Further MR analyses are needed to estimate the causal relationship between TRP and KYN and IBD at different stages.

Second, although our results suggest that TRP plays a protective role for IBD outcomes and KYN is a risk factor for IBD, the results of MR analyses are only based on genetic evidence. Additionally, definitive causal relationships require further mechanistic studies and randomized controlled trials. Third, although the populations we studied were all European, they were not from the same country, which may introduce some bias. Additionally, due to genetic, environmental, and dietary differences between Eastern and Western populations, these results need to be validated in other ethnic groups. Recent epidemiological surveys have shown that non-European populations are becoming new victims of IBD^1,62^. In Asia, the incidence of IBD has increased rapidly in the past 20 years^63^. We performed MR analysis using IBD^13^ and tryptophan^22^ data from East and South Asian populations. However, due to the lack of large sample sizes and high-quality GWAS data in Asian populations, we did not obtain any significant conclusions. Thus, the support of future large-scale GWAS data is needed to conduct relevant MR studies involving other ethnic groups. Fourth, although we matched all selected SNPs with the PhenoScanner database to avoid potential confounding factors and related horizontal pleiotropy, this measure cannot completely eliminate the influence of horizontal pleiotropy because the exact biological functions of many genetic variants are still unknown.

## Conclusions

This MR study provides new genetic evidence for the causal relationship between key metabolites in TRP–KYN pathway (including TRP and KYN) and the risk of IBD (including UC and CD). We recommend giving due attention to TRP supplementation in IBD patients and propose that disease evaluation and assessment in these patients can be conducted by monitoring the KYN/TRP ratio. Further experiments or population-based observational studies are needed to elucidate the potential mechanisms underlying the relationships among TRP, KYN, and IBD and to explore the possibility of targeted therapy for IBD based on the TRP–KYN metabolic pathway.

### Supplementary Information

Supplementary Figure S1.

Supplementary Figure S2.

Supplementary Figure S3.

Supplementary Figure S4.

Supplementary Figure S5.

Supplementary Figure S6.

Supplementary Tables.

## Data Availability

We used publicly available databases, and all data mentioned in the manuscript can be found on the website provided in the article.
