# Supplementary figures and images for: Association between metabolites in tryptophan-kynurenine pathway and inflammatory bowel disease: a two-sample Mendelian randomization
Source: Sci Rep. 2024 Jan 2;14:201. doi: 10.1038/s41598-023-50990-9 (PMC10761717; doi:10.1038/s41598-023-50990-9)

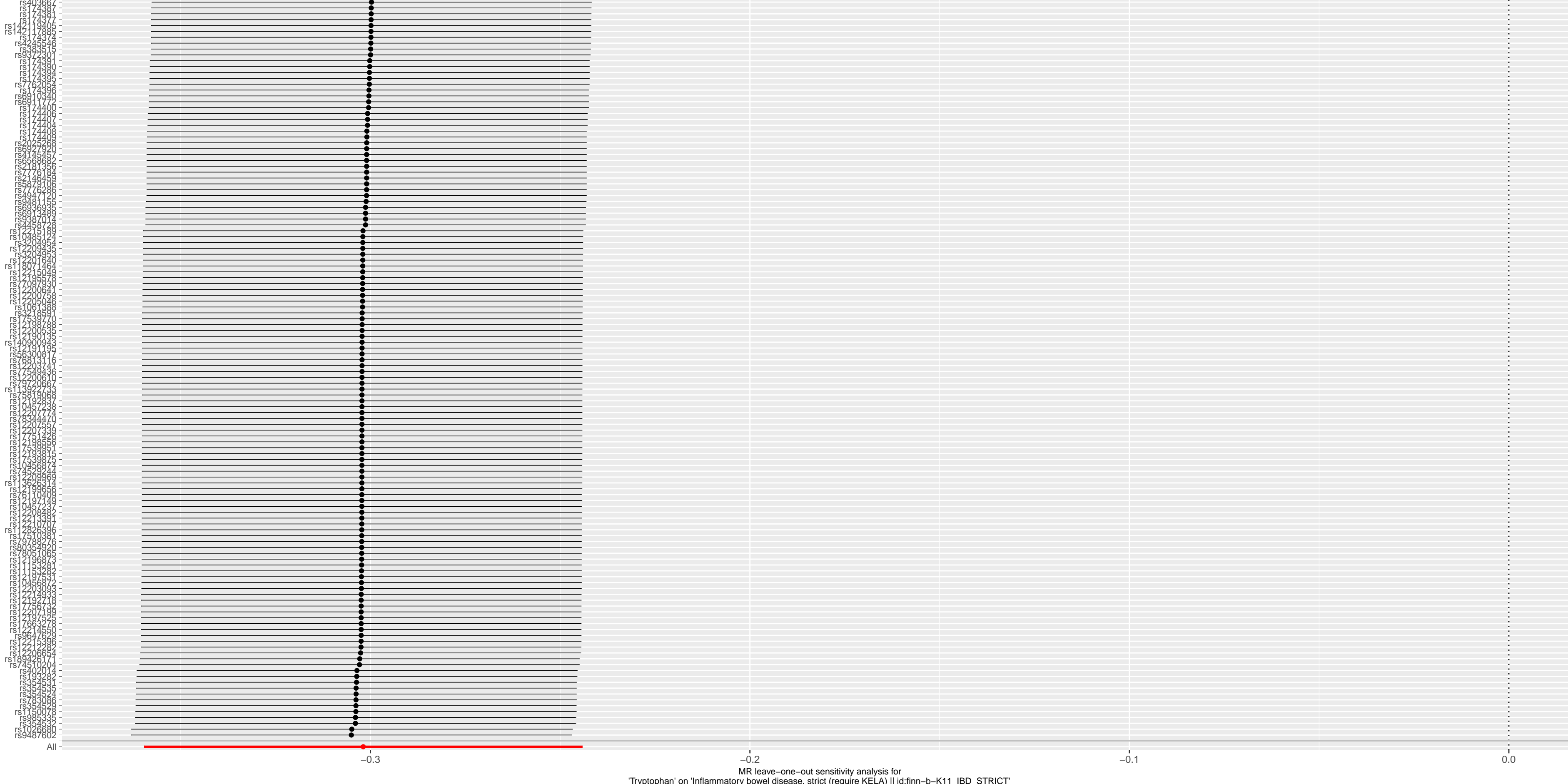

Supplement: Supplementary file 1 — Supplementary Figure S1. [file 41598_2023_50990_MOESM1_ESM.pdf]

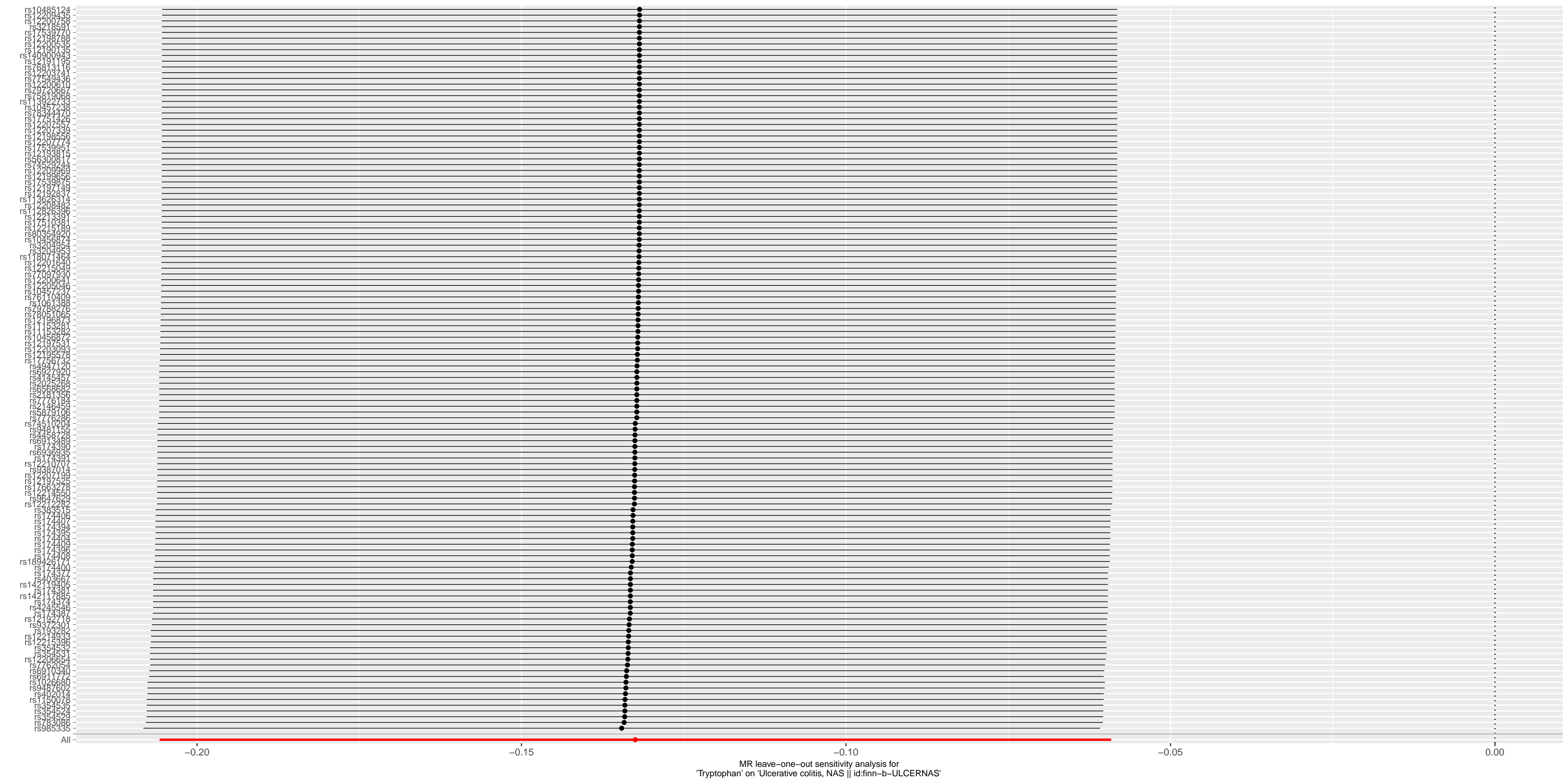

Supplement: Supplementary file 2 — Supplementary Figure S2. [file 41598_2023_50990_MOESM2_ESM.pdf]

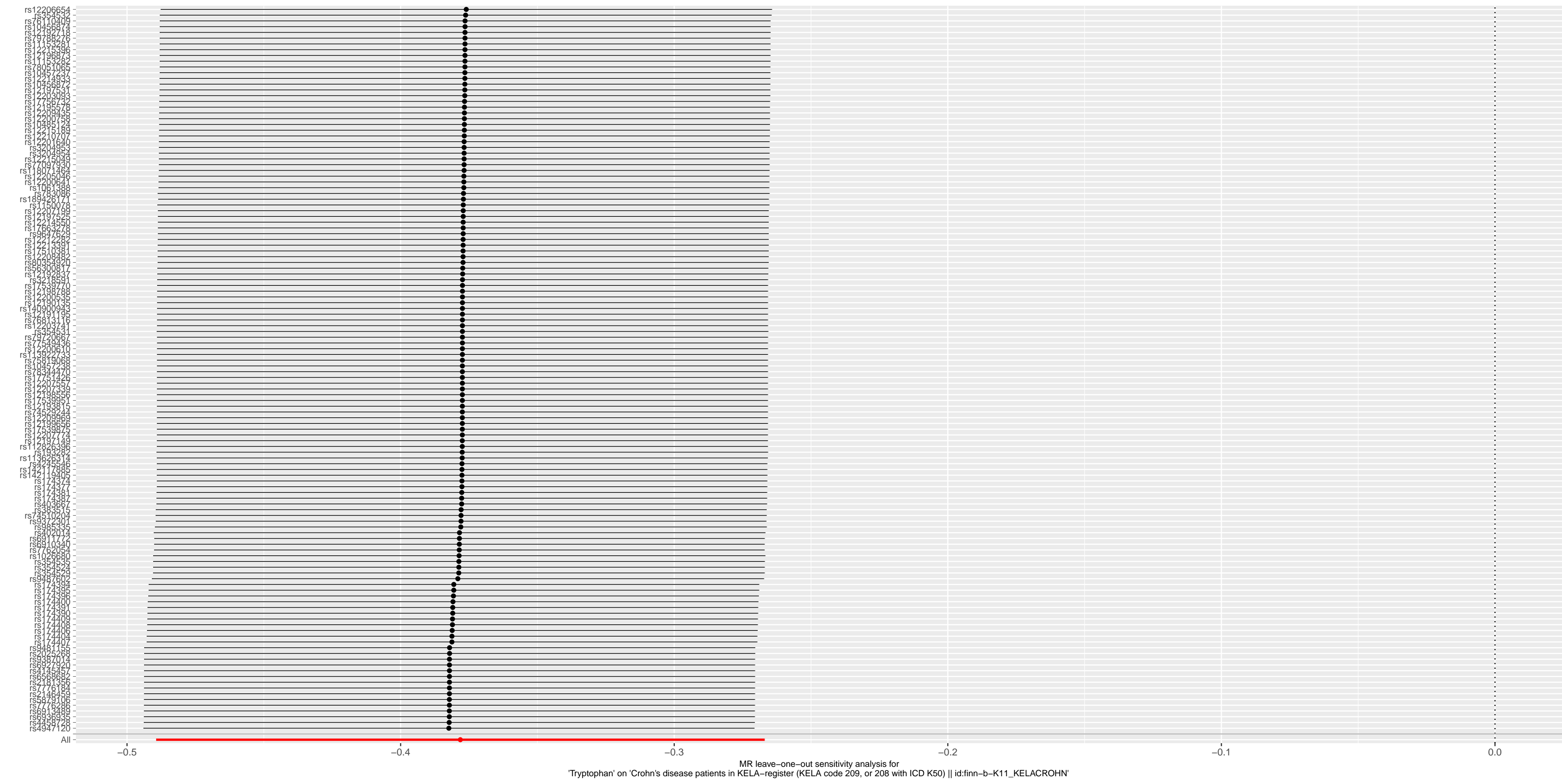

Supplement: Supplementary file 3 — Supplementary Figure S3. [file 41598_2023_50990_MOESM3_ESM.pdf]

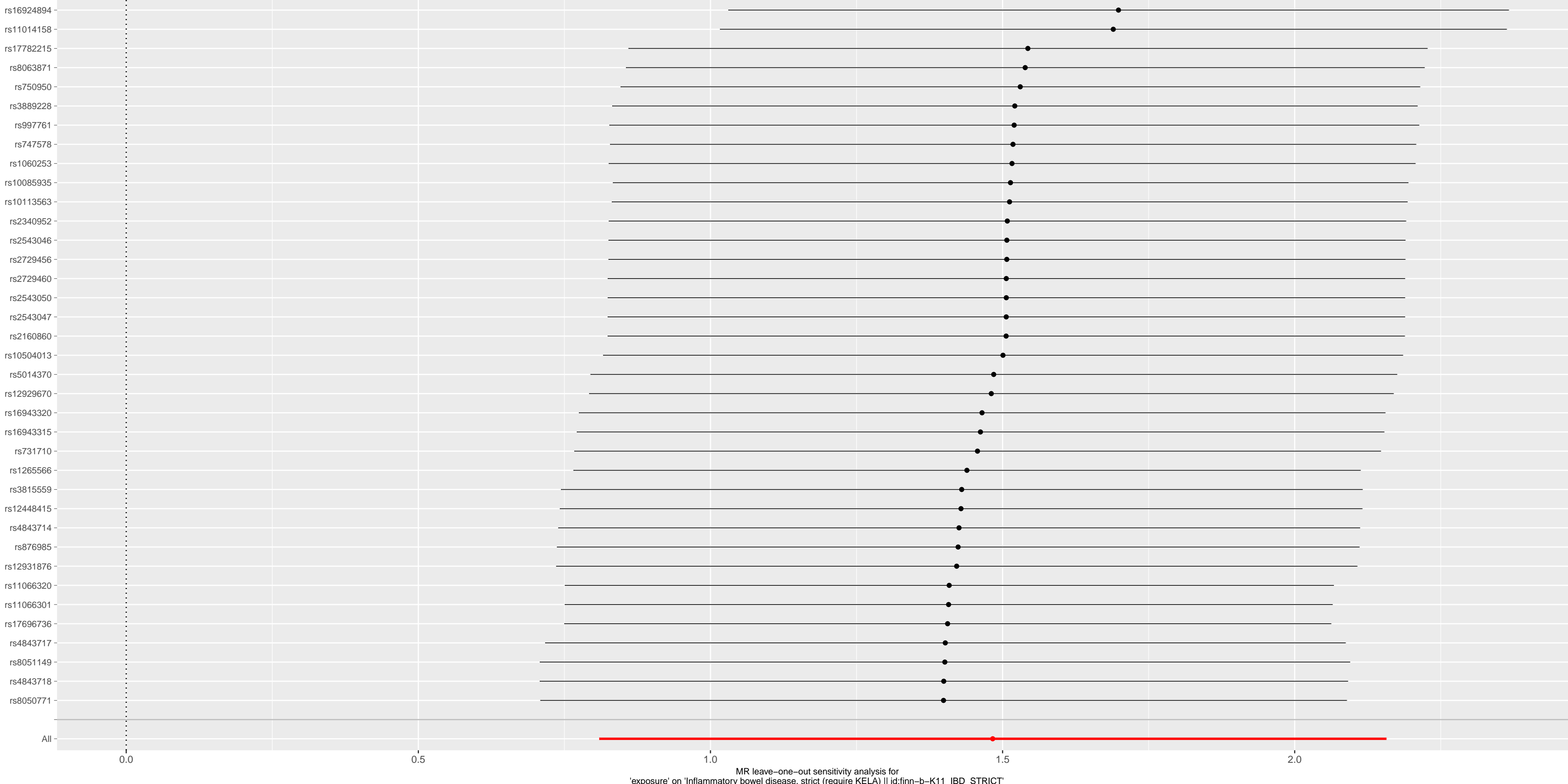

Supplement: Supplementary file 4 — Supplementary Figure S4. [file 41598_2023_50990_MOESM4_ESM.pdf]

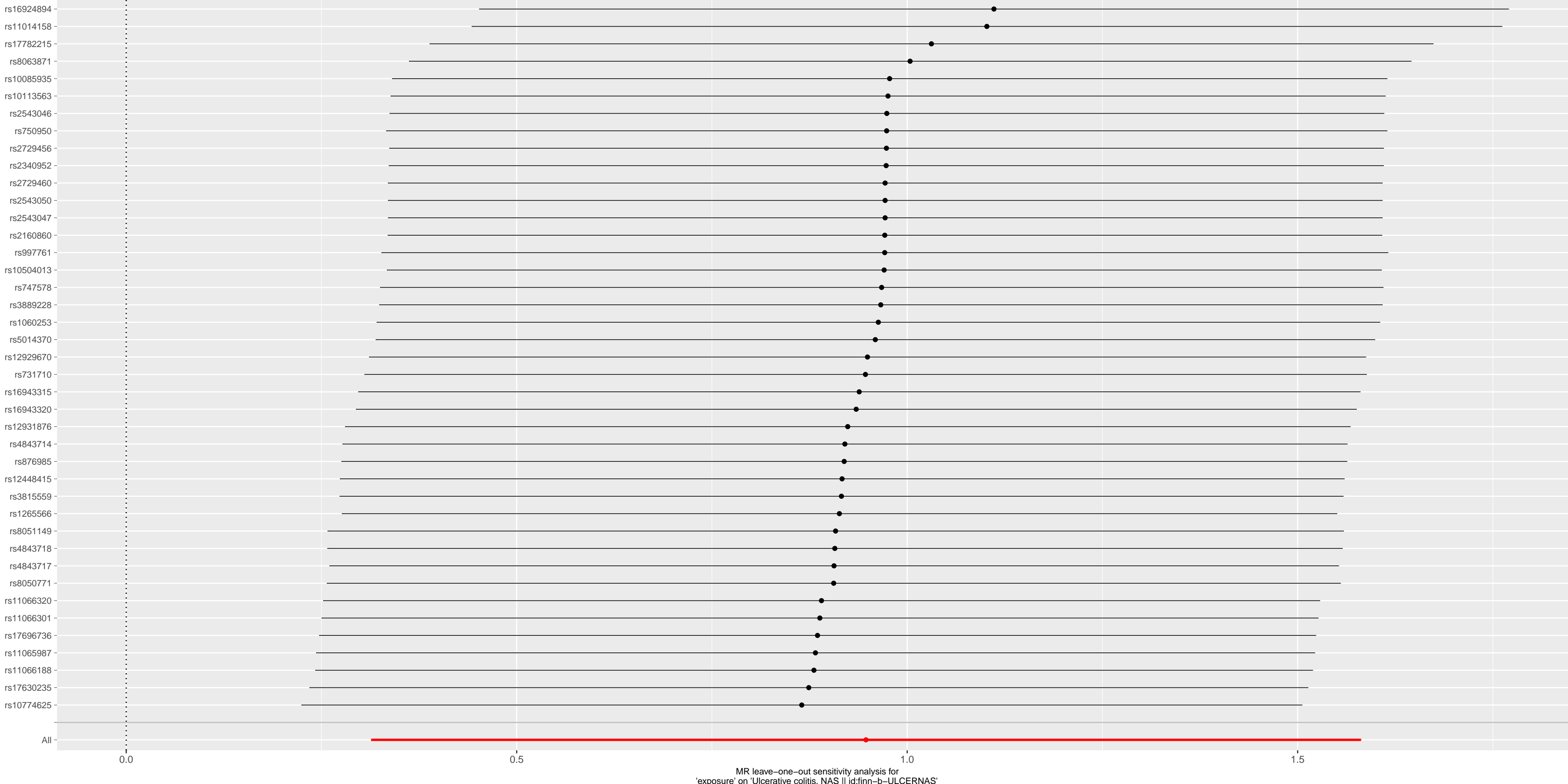

Supplement: Supplementary file 5 — Supplementary Figure S5. [file 41598_2023_50990_MOESM5_ESM.pdf]

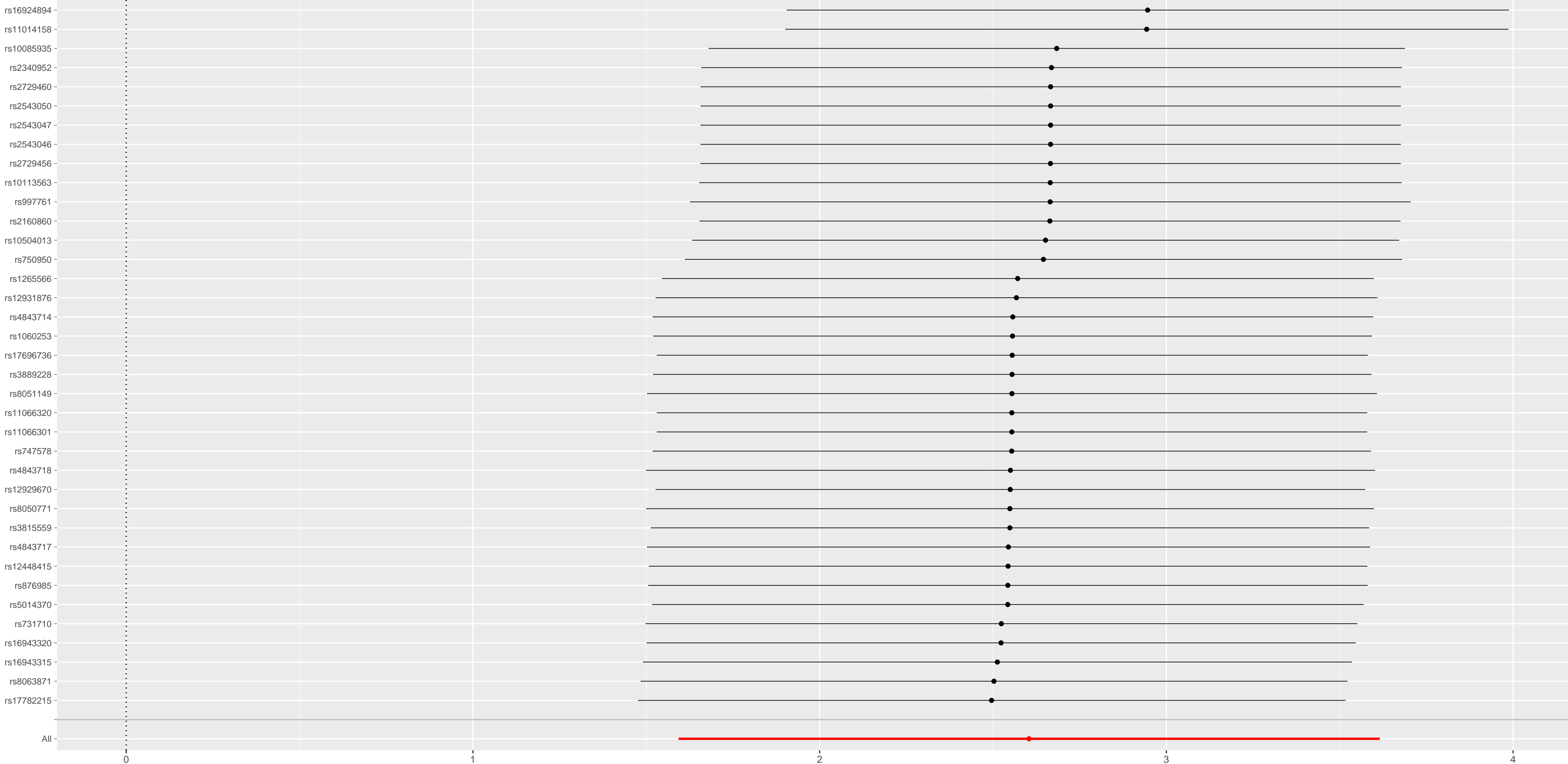

Supplement: Supplementary file 6 — Supplementary Figure S6. [file 41598_2023_50990_MOESM6_ESM.pdf]
